# Editorial: Fish behaviour and welfare

**DOI:** 10.3389/fvets.2024.1462812

**Published:** 2024-09-03

**Authors:** Pablo Almazán-Rueda, Ana C. Puello-Cruz, Rosario Martínez-Yáñez

**Affiliations:** ^1^Centro de Investigación en Alimentación y Desarrollo, AC, Mazatlan, Mexico; ^2^Departamento de Veterinaria y Zootecnia, Universidad de Guanajuato, Irapuato, Mexico

**Keywords:** fish, health, welfare, behavior, aquaculture

World fish production from both aquaculture and fisheries increased in 2022, with a record number of around 223.2 million tons, from which 185.4 million tons consist of aquatic animals and 37.8 million tons are algae (1). Fish culture is an important source for many households in different parts of the world. One of its principal objectives is to effectively apply dry feed (pelleted feed) for promoting fish growth. In 2022, global aquaculture surpassed the 130.9 million tons, of which 94.4 million tons were aquatic animals, 51 percent of the total aquatic animal production. The principal source of fish, as one could see, is aquaculture. Number of fish farms has increased considerably so that the demand of higher population and the need for water related products are met. This has resulted in public concern on how to produce aquaculture products for human consumption, principally on the area of fish welfare. Fish (food) can be produced in an environmentally correct way without causing undue suffering to cultivated organisms. Captivity conditions must contribute to the health and welfare of each animal. Fish must be cared for by trained and experienced personnel, including providing veterinary care, and researchers must be strictly qualified and have sufficient experience and training before undertaking animal research (2). Different approach to different welfare topics of different species are presented in this book. All the different topics have in common the wellbeing of the fish that is being cultured for our benefit: Fish life cycle; from egg to slaughter: monitoring the welfare of Nile tilapia, Oreochromis niloticus, throughout its entire life cycle in aquaculture (Pedrazzani et al.). Welfare indicators under farm conditions: welfare indicators in Tilapia: an Epidemiological Approach (Flores-García et al.), and Qualitative Behavioral Assessments, a welfare indicator for farmed Atlantic Salmon (Salmo salar) in response to a stressful challenge (Wiese et al.). Reproduction: Use of male-to-female sex reversal as a welfare scoring system in the protandrous farmed gilthead sea bream (Spaurus aurata) (Holhorea et al.). Farm conditions: Experimental study on the effect of sound stimulation on hearing and behavior of juvenile black rockfish (*Sebastes schleglii*) (Wang et al.); The effects of aerator noise on the swimming, feeding, and growth of *Micropterus salmoides* (Zhang et al.); and Behavior analysis of juvenile steelhead trout under blue and red light color conditions based on multiple object tracking (Li et al.). Sedation: Does sedation with AQUI-S^®^ mitigate transport stress and post transport mortality in ballan wrasse (*Labrus bergyltae*)? (Calabrese et al.). Killing methods: Farmed fish welfare during slaughter in Italy: survey on stunning and killing methods and indicators of unconsciousness (Clemente et al.); and Humane slaughter in Mediterranean Sea bass and bream aquaculture: farm characteristics, stakeholder views, and policy implications (van Pelt et al.).
